# Use of shotgun metagenomics for the identification of protozoa in the gut microbiota of healthy individuals from worldwide populations with various industrialization levels

**DOI:** 10.1371/journal.pone.0211139

**Published:** 2019-02-06

**Authors:** Ana Lokmer, Amandine Cian, Alain Froment, Nausicaa Gantois, Eric Viscogliosi, Magali Chabé, Laure Ségurel

**Affiliations:** 1 UMR7206 Eco-anthropologie et Ethnobiologie, CNRS—MNHN—Univ Paris Diderot—Sorbonne Paris Cité, Paris, France; 2 Univ. Lille, CNRS, Inserm, CHU Lille, Institut Pasteur de Lille, U1019 –UMR 8204 –CIIL–Centre d’Infection et d’Immunité de Lille, Lille, France; Colorado State University, UNITED STATES

## Abstract

Protozoa have long been considered undesirable residents of the human gut, but recent findings suggest that some of them may positively affect the gut ecosystem. To better understand the role and ecological dynamics of these commensal and potentially beneficial protozoan symbionts, we need efficient methods to detect them, as well as accurate estimates of their prevalence across human populations. Metagenomics provides such an opportunity, allowing simultaneous detection of multiple symbionts in a single analytical procedure. In this study, we collected fecal samples of 68 individuals from three Cameroonian populations with different subsistence modes and compared metagenomics-based and targeted methods of detection for two common protozoan genera: *Blastocystis* and *Entamoeba*. In addition, we analyzed our data along with publicly available fecal metagenomes from various worldwide populations to explore the prevalence and association patterns of ten protozoan genera. Regarding the detection method, microscopy was much less sensitive than metagenomics for *Entamoeba*, whereas qPCR was at least as sensitive as metagenomics for *Blastocystis* sp. However, metagenomics was more likely to detect co-colonizations by multiple subtypes. Out of the ten examined genera in 127 individuals from Cameroon, Tanzania, Peru, Italy or USA, only three (*Blastocystis*, *Entamoeba* and *Enteromonas*) had an overall prevalence exceeding 10%. All three genera were more common in less industrialized populations and their prevalence differed between continents and subsistence modes, albeit not in a straightforward manner. The majority (72.5%) of colonized individuals carried at least two protozoan species, indicating that mixed-species colonizations are common. In addition, we detected only positive and no negative association patterns between different protozoa. Despite the pitfalls of the metagenomic approach, ranging from the availability of good-quality sequencing data to the lack of standard analytical procedures, we demonstrated its utility in simultaneous detection of multiple protozoan genera, and especially its ability to efficiently detect mixed-species colonizations. Our study corroborates and expands prevalence results previously obtained for *Blastocystis* sp. and provides novel data for *Entamoeba* spp. and several other protozoan genera. Furthermore, it indicates that multiple protozoa are common residents of the healthy human gut worldwide.

## Introduction

Gut protozoa have long been exclusively a topic of parasitological research, although they may actually be more often commensal than pathogenic [1, 2]. Recent findings even suggest that some protozoa, such as *Blastocystis* sp. and non-pathogenic *Entamoeba* spp., could benefit their hosts, as they are associated with an increased gut microbiome diversity and a higher frequency of potentially beneficial bacterial taxa [2–6]. It has thus been proposed that the virtual eradication of gut protozoa in industrialized countries might negatively influence human health due to the disruption of ecological interactions in the gut ecosystem [2, 6]. In addition, there is a number of other protozoan species that may affect the gut ecosystem, but whose pathogenicity and prevalence are even less known [2]. In order to study the ecological and clinical impact of gut protozoa, we first need reliable estimates of their prevalence.

To date, the prevalence of gut protozoa is often underestimated due to the lack of adequate detection and surveillance systems [7]. Furthermore, these organisms are often studied separately, impeding the understanding of their ecological dynamics. Finally, traditional diagnostic procedures based on microbiological methods including cultivation, microscopy and antigen-based tests, have significant drawbacks such as a high limit of detection and are available only for a limited number of pathogens. However, a recent shift towards molecular procedures has increased diagnostic sensitivity and specificity, and improved quantification [7]. Notably, it is now recognized that non-pathogenic *Entamoeba* spp. have a non-negligible prevalence in many African populations (0–40%, [8, 9]). Moreover, genotyping of *Entamoeba* spp. revealed that, in Africa, *E*. *dispar* is far more common than *E*. *histolytica* [10]. Differentiating between the pathogenic *E*. *histolytica* and morphologically undistinguishable non-pathogenic *Entamoeba* species, *E*. *dispar* and *E*. *moshkovskii* [10], is of great importance for public health, as *E*. *histolytica* is among the major causative agents of diarrheal disease, especially in developing regions such as sub-Saharan Africa [11]. However, reliable data on the worldwide distribution of *Entamoeba* spp. remain scarce. Similarly, molecular methods unveiled high genetic diversity of *Blastocystis* sp., the most common single-celled eukaryote in fecal samples of healthy humans [12–19]: so far, 10 genetic subtypes (ST1–9 and ST12) have been found in humans, with ST3 being the most common [16]. Apart from *Entamoeba* and *Blastocystis*, other presumably non-pathogenic intestinal protozoa have been studied only exceptionally by molecular methods [1].

Recent accumulation of shotgun metagenomic data from human fecal samples provides an unprecedented opportunity to simultaneously obtain detailed taxonomical and genetic information for a broad range of gut protozoan species, as metagenomics (MG) allows joint identification and genomic characterization of multiple species without the need for species-specific procedures [20, 21]. Another advantage of this method is the ability to identify mixed-species colonizations. Indeed, MG bypasses one of the disadvantages of targeted PCR or qPCR techniques—competitive amplification of differentially abundant strains in a sample.

To date, the few studies that used metagenomics to detect gut protozoa investigated only *Blastocystis* sp. [22–24], likely because they focused on highly industrialized populations with extremely low prevalence of other gut protozoa. To our knowledge, no study has evaluated the potential of MG in the detection of gut protozoa (pathogenic or non-pathogenic) other than *Blastocystis* sp so far.

In our study, we aimed to address these gaps in the field in the following way: i) by assessing the potential of MG for diagnosis of protozoan (co-)colonization; ii) by estimating the prevalence of various gut protozoa in healthy individuals and testing whether they are common residents of a healthy human gut; iii) by exploring the influence of industrialization and sanitary conditions on the diversity and prevalence of gut protozoa; and iv) by examining association patterns (co-occurrence and co-exclusion) among the studied protozoa in order to make predictions about the ecological factors shaping the gut protozoan community.

In order to do so, we first compared the MG-based approach with targeted diagnostic methods (microscopy for *Entamoeba* spp. detection and qPCR for *Blastocystis* sp. STs detection) in 68 Cameroonian adults. We then analyzed the metagenomes generated from these Cameroonian samples together with publicly available metagenomes from Tanzanian, Peruvian, USA and Italian individuals [25, 26], summing up to 127 worldwide fecal metagenomes of healthy individuals. We systematically looked for multiple pathogenic and non-pathogenic protozoa: *Blastocystis* sp., *Chilomastix mesnili*, *Cryptosporidium* spp., *Cystoisospora belli*, *Dientamoeba fragilis*, *Endolimax nana*, *Entamoeba* spp., *Enteromonas hominis*, *Giardia intestinalis* and *Iodamoeba bütschlii*. We took advantage of this combined dataset to assess the prevalence of gut protozoa in populations with varying levels of industrialization (rural populations with traditional modes of subsistence, further referred to as non-industrialized populations, and urban industrialized populations). We also examined the effect of subsistence mode (hunter-gatherer, farmer or fisher) on gut protozoa prevalence in non-industrialized populations. Although we do not believe that diet is a major determinant of gut eukaryotes diversity, we hypothesize that there are likely systematic sanitary differences between these subsistence modes that result in varying environmental exposure to gut eukaryotes. Finally, as we simultaneously determined the presence of multiple non-pathogenic and pathogenic gut protozoa in the studied individuals, we were able to investigate (to our knowledge for the first time) association patterns between them.

## Material and methods

### Ethics statement

The research protocol was approved by the CPP (Comité de Protection des Personnes) French ethical committee (N°2010-avril-12276) and samples were authorized to be collected and conserved by the French Ministry of Higher Education and Research (N°DC 2009–1068). Research permits were obtained by the “Institut de Recherche pour le Développement “(IRD) in agreement with the “Ministère de la Recherche Scientifique et de l’Innovation” (MINRESI) of Cameroon. In addition, an ethical clearance was obtained from the Cameroonese National Committee (CNERSH, Central National d’Ethique de la Recherche pour la Santé Humaine, Approval N 2017/05/900) and a regional research permit was obtained from the Health Minister (Centre region, Approval N°0061). The CPP (Comité de Protection des Personnes) clearance was required the Cameroon national ethical committee for the application submission. Written consent was obtained from all participants.

### Sample collection

Fecal samples were collected from 68 healthy individuals (23 hunter-gatherers, 24 farmers and 21 individuals from a fishing population; 40 males and 28 females) aged 26 to 78 years (median of 50 years) and living in seven different villages situated in Southwest Cameroon (see [3]). Informed consent for this research project was obtained from all volunteers sampled in this study. The 16S-rRNA-gene based characterization of these samples including the contextual data has been previously published [3].

### Metagenomic data

Metagenomic data were obtained for 57 of the 68 sampled individuals from Cameroon. The DNA Libraries were prepared using Nextera XT DNA Library Prep Kit (Illumina, San Diego, CA, USA) and sequenced as 100 bp paired-end reads on a HiSeq2000 sequencer (Illumina) at the University of Minnesota Genomics Center (Minneapolis, MN, USA). Raw data have been deposited in the European Nucleotide Archive (ENA) with the BioProject ID PRJEB27005, under the accession numbers ERS2539904-ERS2539960. We obtained an average of 25.6 million read pairs per sample. The reads from low-quality areas of the flowcell were filtered out using FilterByTile tool from BBMap package [27].

We further explored metagenomic datasets focusing on non-industrialized populations published by Obregon-Tito et al., Rampelli et al. and Yatsunenko et al. [25, 26, 28], which we downloaded from SRA NCBI and MG-RAST in June 2017. However, we did not pursue with the Yatsunenko dataset (rural individuals from Venezuela and Malawi, and urban individuals from USA) due to their low sequencing depth (average of 155,890 350bp reads per sample) [28]. The Rampelli dataset (study accession: PRJNA278393; average of 10.9 million 100bp read pairs per sample) [26] consists of 27 healthy rural Hadza of Tanzania and 11 healthy urban Italians. The Obregon-Tito dataset (study accession: PRJNA268964, a mix of 75bp, 100 bp and 150 bp paired-end reads with an average of 22.3 million read pairs per sample) [25] consists of 12 rural Tunapuco and 24 rural Matses healthy individuals from Peru and 22 urban healthy subjects from Norman, USA. As all Cameroonian subjects were adults, we removed the individuals younger than 18 years of age from the two downloaded datasets to increase comparability, resulting in 22 Hadza, 11 Italians, 8 Tunapuco, 10 Matses, and 19 adults from Norman. Details about the samples used, together with the number of reads per sample, can be found in S1 Table.

For all datasets, we used the bbduk tool [27] to trim the adapters, very low-quality bases (Phred Q<3) and to remove reads shorter than 36 bp. The quality of the metagenomic data was assessed by FastQC [29].

### Detection of various gut protozoa using metagenomic data

We searched for multiple gut protozoa species, i.e., *Blastocystis* sp., *Chilomastix mesnili*, *Cryptosporidium hominis*, *Cryptosporidium parvum*, *Cystoisospora belli*, *Dientamoeba fragilis*, *Endolimax nana*, *Entamoeba* spp., *Enteromonas hominis*, *Giardia intestinalis* and *Iodamoeba bütschlii*, by aligning quality-controlled data to publicly available data using BWA-MEM [30]. The SSU rRNA gene sequences of *Retortamonas* and *Pentatrichomonas* available in public databases were either very short or not of human origin and therefore they were not included in the analysis. To detect *Blastocystis* sp., *Giardia intestinalis*, *Cryptosporidium hominis* and *C*. *parvum*, we used whole-genome data, while, for the other species (including all *Entamoeba* spp.), we used complete SSU rRNA gene sequences (S2 Table). For *E*. *histolytica* and *E*. *dispar* (for which both genomes and complete SSU sequences are available), we decided to use the SSU rRNA gene sequences, in order to obtain comparable data for a larger number of *Entamoeba* species.

In order to retain only high-confidence alignments for the species with available genomes, we applied the following filtering procedure. We used SAMTools [31] to remove the reads with unmapped mates (-F8) and supplementary alignments (-F2048). We further used an in-house script to sequentially filter out the alignments with more than 0.1 of the read length soft-clipped, with edit distance exceeding 0.01 of the read length and finally with the alignment length shorter than 75 bp. As genomes from public databases can contain vectors and sequencing artifacts, we performed an additional step to remove the alignments to such regions. In detail, we extracted the sequences of the hit genomic regions with BEDTools [32] and excluded those that mapped with high confidence (utilizing the similar procedure as above with adjusted cut-offs: 0.02 of the read length for edit distance, and alignment length > 35 bp) to any of 120 bacterial reference genomes [33], UniVec database [34] or to the "sequencingartifacts" file provided by BBmap [27]. To further decrease the rate of false positives due to similarity between the genomes, we removed the reads that mapped to multiple genomes from further analysis. However, this step had no effect on the results (results not shown).

For the species with SSU rRNA gene sequences, we performed a similar procedure with the following adjustments: we did not require both reads to map and we applied a 0.01 read length cutoff for soft-clipping (instead of 0.1). We additionally removed all read pairs where each mate mapped to different species to reduce the false positive rate. As no reads mapped uniquely to *E*. *histolytica*, we excluded it from final analysis in order to correctly estimate the abundance of *E*. *dispar* (which has a highly similar SSU rRNA sequence).

In case of SSU rRNA gene sequences, we assumed that any hit passing the above filtering criteria could be considered reliable and we therefore used the presence of hits as our diagnostic. For whole genomes, we additionally inspected the distribution of hits across the scaffolds and calculated the breadth of coverage. Namely, shotgun reads may map to contaminating sequences (such as vectors or sequences originating from other organisms) that publicly available genomes often contain, which may result in a high number of hits concentrated in few regions and very low breadth of coverage. This is indeed what we observed in few cases, primarily for *Blastocystis* sp. ST6 (S5 Fig).

### qPCR and molecular subtyping of *Blastocystis* sp. in Cameroon

For 68 samples from Cameroonian subjects, 1 μl of extracted DNA was subjected to a qPCR assay using the *Blastocystis* sp.-specific primers BL18SPPF1 (5’-AGTAGTCATACGCTCGTCTCAAA-3’) and BL18SR2PP (5’-TCTTCGTTACCCGTTACTGC-3’) targeting the SSU rRNA gene as previously described [35]. The positive qPCR products were purified and both strands were directly sequenced at Genoscreen, Lille, France. Direct sequencing of several qPCR products generated mixed signals that could reflect colonization by different STs. These samples were thus re-analyzed by non-qPCR using the same primer pair as for qPCR, followed by cloning. In detail, end-point PCR amplifications were performed in 50 μl according to standard conditions for Platinum *Taq* High-Fidelity DNA polymerase (Invitrogen, Groningen, the Netherlands). After denaturation at 94°C for 5 min, 40 cycles of amplification were performed with a Bioer LifeECO apparatus (Binjiang District, China) as follows: 30 s at 94°C, 35 s at 60°C, and 50 s at 68°C. The final extension was continued for 2 min. End-point PCR products were separated by agarose gel electrophoresis and bands of the expected size (approximately 320 bp) were purified using the Wizard SV Gel and PCR clean-up system (Promega, Madison, WI, USA). Purified PCR products were cloned in the T-vector, pCR 2.1-TOPO (Invitrogen) and amplified in *Escherichia coli* One Shot TOP10 competent cells. Minipreparations of plasmid DNA were done using the NucleoSpin Plasmid kit (Macherey-Nagel, Düren, Germany). Five positive clones containing inserts of approximately the expected size were arbitrarily selected for each sample and sequenced in both directions. The sequences obtained were compared with all *Blastocystis* sp. homologous sequences available from the National Centre for Biotechnology Information (NCBI) using the nucleotide BLAST program. The STs were identified by finding the exact or closest match against all known mammalian and avian *Blastocystis* sp. STs according to the last classification by Alfellani et al. [36]. All sequences shared 99–100% identity with the publicly available sequences, allowing the direct subtyping of the corresponding isolates. The sequences were deposited in GenBank and are available under accession numbers MG907124-MG907188.

### Statistical tests

All statistical analyses were conducted in R [37]. We compared different methods of detection with McNemar's test and we calculated a corresponding odds ratio with 95% confidence intervals to estimate the effect size if applicable. To examine the differences between populations, we performed Fisher’s exact tests, first grouping non-industrialized and industrialized populations together, and then examining differences among non-industrialized populations only. If the latter was significant, we fitted a logistic regression model to examine if these differences were due to the country of origin and/or to subsistence mode. The particular contrasts we were interested in were between: Africa and South America, Eastern and Western Africa (*i*.*e*. Tanzania and Cameroon), hunter-gatherers and farmers/fishers, and between farmers and fishers. As logistic regressions do not allow for reliable estimation of coefficients and their confidence intervals if prevalence is 0%, we excluded the contrasts where all populations of one group had 0% prevalence.

We tested if *Entamoeba* spp. and *Blastocystis* sp. preferentially infect the same individuals with a Chi-square test. We additionally calculated a Pearson’s phi coefficient to provide an estimate of effect size where appropriate. We further looked for significant association patterns between each pair of protozoan species/STs by conducting co-occurrence analysis implemented in the R-package *cooccur* [38]. Observed and expected frequencies of co-occurrence between each pair of species are calculated with the expected frequencies assuming random and independent distribution of each ST/species. The analysis returns the probabilities that a more extreme (either low or high) value of co-occurrence could have been obtained by chance. Coinfection plots were created with the R-package *UpSetR* [39].

## Results

### Detection of gut protozoa from metagenomic data

In order to detect gut protozoan species belonging to ten different genera and estimate their prevalence in eight populations across four continents, we mapped 127 gut metagenomes (from 57 individuals living in Cameroon, 22 in Tanzania, 18 in Peru, 19 in USA and 11 in Italy) either to available protozoan genomes or to SSU rRNA gene sequences (listed in S2 Table). To keep the false positive rate as low as possible, we filtered the alignments in a stepwise manner, applying strict filtering criteria (see Methods section for details). Briefly, our diagnostic measure for genome-based detection relied on two criteria: we first selected potential positives based on the percentage of positive contigs and subsequently excluded cases with breadth of coverage < 0.001. We chose the percentage of positive contigs over the breadth of coverage as a primary criterion because the latter may vary more widely depending on the size of contaminating genome regions (i.e. not originating from the protozoan itself) than the distribution of hits across the contigs. Still, the number and size of contigs/scaffolds depends on the assembly quality, and the assemblies with very few contigs could easily result in false positives without the second step.

We observed a uniform-like hit distribution in the samples with at least 5% positive contigs. Applying a 5% or 10% detection threshold yielded highly concordant results for *Blastocystis* sp. (McNemar’s chi-square (1) = 1.33, p = 0.248, N = 127), with the same STs detected using both thresholds. In addition, the number of positive individuals was fully concordant between these thresholds for ST1 and did not significantly differ for ST2 and ST3 (McNemar’s test: ST2: chi-square (1) = 2.25, p = 0.134; ST3: chi-square (1) = 0.5, p = 0.480). We decided to be stringent and use 10% positive contigs as our diagnostic threshold in the rest of the work.

For the analysis of SSU rRNA gene sequences, we used the presence of hits (after the alignment filtering step, see details in Methods) as a diagnostic measure. We did not detect *Entamoeba histolytica*, *Entamoeba chattoni*, *Cryptosporidium hominis*, *C*. *parvum* or *Blastocystis* ST6, ST7 and ST9 in any of the 127 samples. All other species had at least one positive individual (S3 Table). Although 71.7% of individuals carried at least one protozoan, the only genera with an overall prevalence exceeding 10% were *Blastocystis* (60.6%), *Entamoeba* (45.7%) and *Enteromonas* (11.0%), and these were thus the only ones analyzed in detail.

### Comparison of detection methods for *Blastocystis* sp. and *Entamoeba* spp. in Cameroon

**Fig 1:**
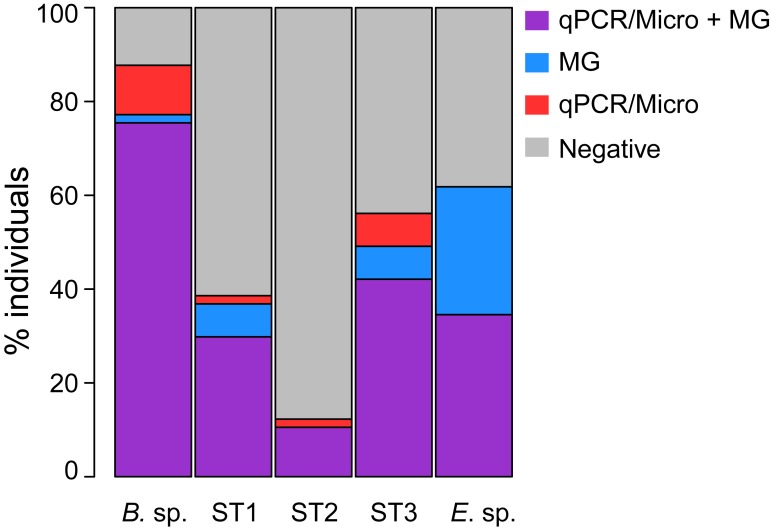
Concordance between detection methods for *Blastocystis* sp. (overall and by subtype, N = 57) and *Entamoeba* spp. (N = 55) in Cameroon. Shown are the proportions of individuals positive for both methods (MG-based and targeted approach), only one of them, or none. *Blastocystis* sp. (*B*. sp.) was assessed by MG and qPCR, while *Entamoeba* spp. (*E*. sp.) was assessed by MG and microscopy (Micro).

To compare the MG-based approach with targeted diagnostic methods, the Cameroonian samples were analyzed by qPCR (*Blastocystis* sp.) and microscopy (*Entamoeba* spp.) in addition to MG. The overall prevalence of *Blastocystis* sp. was 88.2% according to qPCR (N = 68) and 77.2% according to MG (N = 57). We obtained congruent results among the 57 samples analyzed by both methods (McNemar’s chi-square (1) = 2.286, p = 0.131), with 75.4% of individuals identified as positive and 12.3% as negative by both of them (Fig 1). Regarding the remaining cases, 10.5% were positive only by qPCR and 1.8% only by MG. Therefore, although we found no statistical difference between qPCR and MG methods for detection of *Blastocystis* sp., qPCR seems a bit more sensitive.

Regarding the distribution of *Blastocystis* sp. STs, both methods detected single colonization by ST3 as the most prevalent (42.6% and 31.6% by qPCR and MG, respectively), followed by ST1 (27.9% and 21.1%) and ST2 (10.3% and 5.3%) single colonizations (Fig 1). Mixed-subtype colonizations were found in 7.4% (qPCR) and 19.3% (MG) of individuals, indicating that MG is more likely to detect these. The combination of ST1/ST3 accounted for the majority of mixed colonizations (60% and 73% by qPCR and MG, respectively), followed by ST2/ST3 (20% and 18%) and ST1/ST2 (20% and 9%). The results were concordant between the methods for each of the three STs (Fig 1, McNemar’s test: ST1: chi-square (1) = 0.8, p = 0.371; ST2: chi-square (1) = 0, p = 1; ST3: chi-square (1) = 0, p = 1).

For the prevalence of *Entamoeba* spp., microscopy yielded a considerably lower estimate (37.9%, N = 66) than MG (63.2%, N = 57). The inspection of the 55 samples analyzed by both methods revealed that no sample was positive by microscopy only (Fig 1) and confirmed a significantly higher sensitivity of MG (McNemar’s chi-square (1) = 13.067, p < 10^−3^, odds ratio (OR) with 95% confidence intervals (CI) = 0 [0, 0.34]). As microscopy does not allow for finer taxonomical resolution, we could not compare the distribution of individual *Entamoeba* spp. species between the methods.

### Prevalence of *Blastocystis* sp. in culturally diverse populations

**Fig 2:**
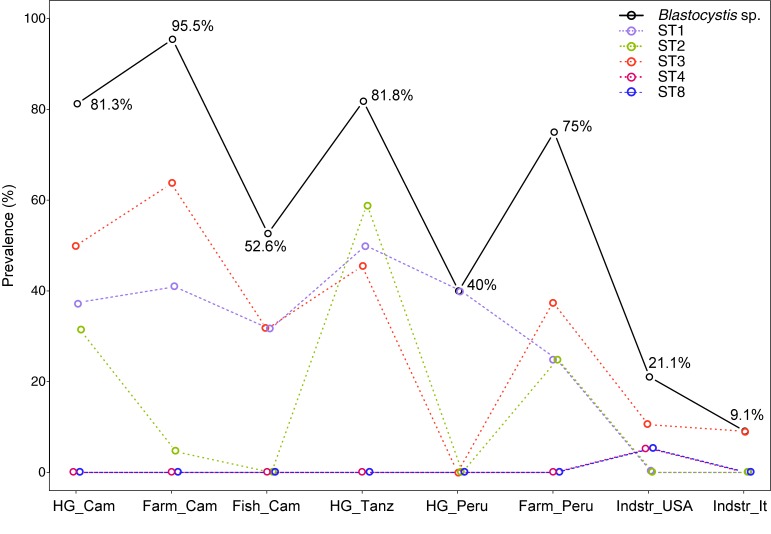
Prevalence of *Blastocystis* sp. and its subtypes across populations according to metagenomic analysis. Subtypes with zero prevalence (ST6, ST7 and ST9) are not shown. Population labels are created as subsistence_country, with following abbreviations: HG = hunter-gatherers, Farm = farmers, Fish = fishers, Indstr = industrialized, Cam = Cameroon, Tanz = Tanzania, It = Italy.

Using MG as the detection method, we found that at least one *Blastocystis* sp. ST was present in 77 out of 127 (60.6%) individuals in the eight studied populations (rural populations: hunter-gatherers, farmers and fishers in Cameroon, hunter-gatherers and farmers in Peru, hunter-gatherers in Tanzania; urban populations: USA and Italy), with the frequency per population ranging from 9.1% in Italy to 95.5% in Cameroonian farmers (Fig 2, S3 Table). Regarding the distribution of *Blastocystis* sp. STs (S1 Fig), the three most common types of colonization were: a single colonization by ST3 (18.1% overall, found in all populations except Peruvian and Tanzanian hunter-gatherers), a single colonization by ST1 (15%, in all African and South-American populations), and a combination of these two STs (9.5%, in Tanzanian hunter-gatherers, Peruvian farmers, Cameroonian farmers and fishers). ST2 was found more often in mixed (11%) than in single colonization (5.5%). Whereas single ST2 colonization was found in African hunter-gatherers as well as in Cameroonian and Peruvian farmers, ST2/ST3 (5.5%) and ST1/ST2 (3.9%) combinations were restricted to African hunter-gatherers. Moreover, mixed-subtype colonization by ST1/ST2/ST3 (1.6%) were detected only in Tanzanian population. ST4 and ST8 were found only in USA, each in one individual.

Considering the influence of subsistence mode on the prevalence of *Blastocystis* sp. (Fig 2, S3 Table), we found a significantly higher prevalence in non-industrialized than in industrialized populations (Fisher's exact test, p < 10^−3^), as well as significant differences between non-industrialized populations (Fisher's exact test, p = 0.004). A logistic regression model including only non-industrialized populations revealed significant effects of both subsistence mode (i.e., farmer, fisher or hunter-gatherer) and country (Likelihood Ratio Test (LRT) for single term deletion, p = 0.013 and 0.001, S4 Table). Specifically, we detected a higher prevalence in Africa than in South America (p = 0.004, OR with 95% CI = 1.89 [1.24, 2.39]) and a higher prevalence in farmers compared with fishers (p = 0.001, OR = 4.28 [1.89, 7.5]).

Regarding the *Blastocystis* sp. STs (Fig 2), ST1 was found only (and in all) non-industrialized countries (Fisher's exact test comparing non-industrialized and industrialized populations, p < 10^−3^), with prevalence ranging from 25% in Peruvian farmers to 50% in Tanzanian hunter-gatherers, but without significant differences between non-industrialized populations (Fisher's exact test, p = 0.836). ST2 was also found only in non-industrialized countries (Fisher's exact test comparing non-industrialized and industrialized populations, p = 0.004), again with the highest prevalence in Tanzanian hunter-gatherers (59.1%). The prevalence of ST2 further significantly differed among non-industrialized populations (Fisher’s exact test, p < 10^−3^). Logistic regression revealed a significant effect of country (LRT = 8.65, p = 0.013), with a higher prevalence in Tanzania than in Cameroon (p = 0.024, OR = 0.44 [0.21, 0.87]), whereas subsistence mode was marginally significant (LRT = 6.33, p = 0.042), likely due to a complete absence of ST2 in fishers (S4 Table). Finally, the most prevalent *Blastocystis* subtype—ST3—was found in all populations except Peruvian hunter-gatherers, with a prevalence ranging from 9.1% in Italy to 63.6% in Cameroonian farmers (Fig 2). Fisher’s exact tests and logistic regression matched the results obtained for overall *Blastocystis* prevalence, reflecting the higher prevalence in non-industrialized countries (Fisher’s exact test, p < 10^−3^) as well as the effect of country (LRT = 9.29, p = 0.010) and subsistence mode (LRT = 6.57, p = 0.037), with significant differences between Africa and South America (p = 0.008, OR = 1.91 [1.23, 3.27]) and farmers and fishers (p = 0.019, OR = 2.16 [1.16, 4.26], S4 Table).

### Prevalence of *Entamoeba* spp. in culturally diverse populations

**Fig 3:**
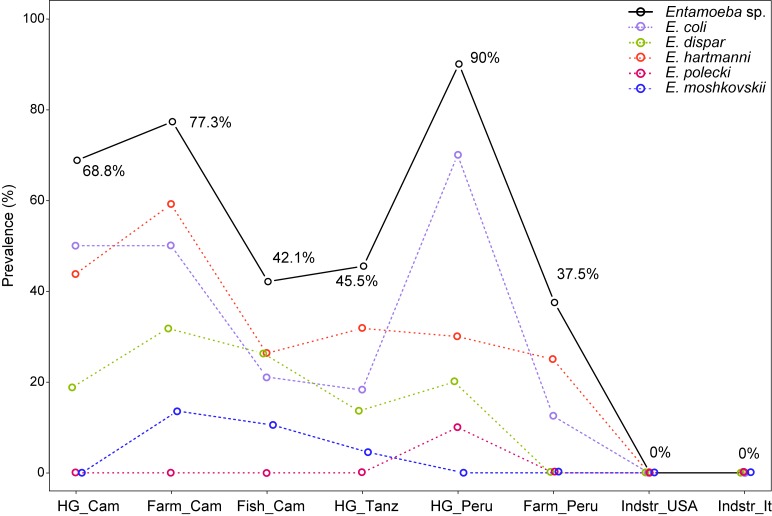
Prevalence of *Entamoeba* spp. and its species across populations according to metagenomic analysis. Species with zero prevalence (*E*. *chattoni* and *E*. *histolytica*) are not shown. The population abbreviations are the same as in Fig 2.

MG-based detection revealed the presence of at least one *Entamoeba* species in 58 out of 127 (45.7%) individuals worldwide, with overall frequencies per population ranging from 0% in Italy and USA to a minimum of 37.5% in non-industrialized populations and reaching up to 90% in Peruvian hunter-gatherers (Fig 3, S3 Table). Regarding the distribution of different *Entamoeba* species within individuals (S2 Fig), although single colonization by either *E*. *coli* or *E*. *hartmanni* were the most common types of colonization (both 10.2%), both species were more often co-occurring with other species: 62.9% and 64.9% of individuals carrying *E*. *coli* resp. *E*. *hartmanni* also carried at least one additional species of *Entamoeba*. Similarly, *E*. *dispar* was found alone only in 2.3% of cases, compared to 12.6% in mixed-species colonizations. Regarding the mixed-species colonizations, the most common one was *E*. *coli/E*. *hartmanni* (8.7%), followed by *E*. *dispar/E*. *hartmanni* and *E*. *coli/E*. *dispar/E*. *hartmanni* (3.1% each), and *E*. *coli/E*. *dispar* (2.4%). The rest of the cases included either *E*. *moshkovskii* (4.7%) or *E*. *polecki* (a single individual, co-infected by *E*. *coli* and *E*. *hartmanni*), both of them found exclusively in mixed-species colonization. Overall, single colonization by *E*. *coli* and *E*. *hartmanni* were found in all non-industrialized populations, whereas all other combinations were restricted to different subsets of these populations.

Considering the prevalence of *Entamoeba* according to subsistence mode (Fig 3, S3 Table), both the difference between non-industrialized and industrialized countries (p < 10^−3^) and the difference among the non-industrialized populations (p = 0.019) were supported by Fisher’s exact test. Logistic regression showed a marginally significant effect of subsistence mode (LRT = 6.23, p = 0.044, S5 Table), reflecting a trend towards higher prevalence in hunter-gatherers compared with farmers and fishers (p = 0.054, OR = 1.46 [1.01, 2.19]), but also in farmers compared with fishers (p = 0.075, OR = 1.76 [0.95, 3.34]). Although the main effect of country was not significant (LRT = 5.53, p = 0.063), regression coefficients suggested higher prevalence in Cameroon than in Tanzania (p = 0.024, OR = 2.18 [1.13, 4.45]).

Regarding the prevalence of individual *Entamoeba* species (Fig 3), Fisher's exact test revealed significantly higher frequencies in non-industrialized than in industrialized populations for *E*. *coli* (p < 10^−3^), *E*. *hartmanni* (p < 10^−3^) *and E*. *dispar* (p = 0.004), but not for *E*. *moshkovskii* nor *E*. *polecki* (p = 0.334 and 1). Only the prevalence of *E*. *coli* significantly varied among non-industrialized populations (Fisher’s exact test, p = 0.013, other species p > 0.188), with differences related to both country and subsistence mode according to the logistic regression model (LRT = 8.48 and 6.57, p = 0.014 and 0.037, respectively; S5 Table). In particular, whereas there was no difference between continents (p = 0.276), the prevalence was higher in Cameroon than in Tanzania (p = 0.007, OR = 2.64 [1.35, 5.65]), and in hunter-gatherers compared with farmers and fishers (p = 0.021, OR = 1.52 [1.07, 2.21]).

### Prevalence of *Enteromonas hominis* in culturally diverse populations

Using MG, we detected *Enteromonas hominis* in all hunter-gatherer populations (6.2% - 50%), as well as in Cameroonian fishers (15.8%), whereas all other populations were negative (S3 Fig, S3 Table). Significant differences in the prevalence of *E*. *hominis* between non-industrialized and industrialized populations as well as within non-industrialized populations were supported by Fisher’s exact test (p = 0.039 and 0.004, respectively). Logistic regression revealed a significant effect of country (LRT = 6.75, p = 0.034), with a higher prevalence in South America than in Africa (p = 0.022, OR = 0.52 [0.28, 0.89]), as well as a significant effect of subsistence mode (LRT = 12.32, p = 0.002), likely reflecting the absence of *E*. *hominis* in farmers (S6 Table). It is noteworthy that *E*. *hominis* was only identified in mixed-species colonization with other protozoa (S4 Fig)).

### Association patterns (co-occurrence and co-exclusion) between protozoan species

Overall, 72.5% of all colonized individuals carried at least two protozoan species and only *Blastocystis* sp. ST1, ST2, ST3 and ST8, *E*. *hartmanni*, *D*. *fragilis* and *C*. *belli* were found alone (S4 Fig). The most prevalent protozoa identified in this study were *Blastocystis* sp. (60.6%) and *Entamoeba* spp. (45.7%). We found that the individuals carrying one of these two genera were more likely to carry the other as well (Chi-square test: chi-square (1) = 17.080, p < 10^−3^, Pearson's phi with 95% CI: 0.383 [0.224, 0.522], df = 125). However, this positive correlation disappears if low-prevalence industrialized populations are excluded (Chi-square test: chi-square (1) = 2.666, p = 0.103), indicating that the above result is mainly due to double-negatives observed in these populations.

**Fig 4:**
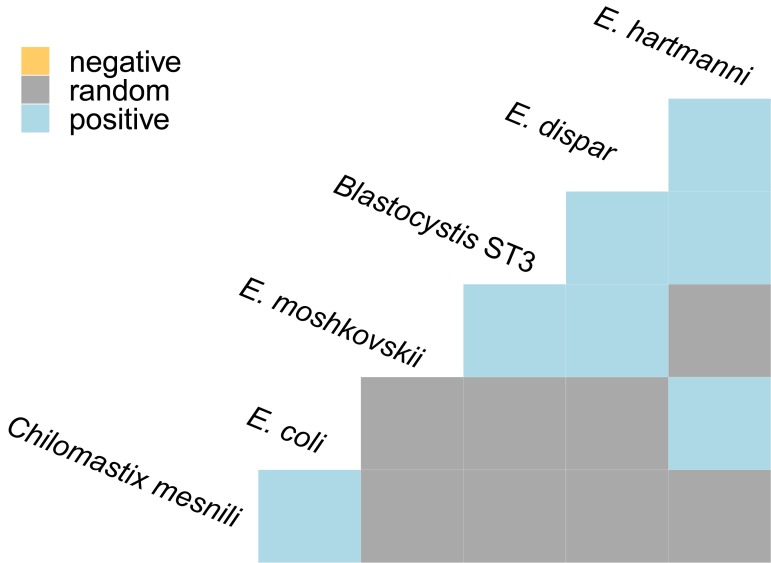
Association patterns between protozoan species in non-industrialized populations. Only species with at least one significant correlation are shown. *E*. *= Entamoeba*.

To examine association patterns between all 17 protozoan species/STs detected in this study, we performed a co-occurrence analysis within non-industrialized populations only. Out of possible 40 pairwise combinations, we detected seven significant associations in six species (Fig 4), all of them positive. Specifically, the most common *Blastocystis* subtype, ST3, was positively correlated with *E*. *dispar*, *E*. *hartmanni* and *E*. *moshkovskii*. Whereas there were no significant associations between the *Blastocystis* sp. STs, *Entamoeba* spp. tended to preferentially occur together. *E*. *coli* was additionally positively associated with *C*. *mesnili*.

## Discussion

### MG: A method for detection and diagnosis of gut protozoa?

Gut protozoa can strongly affect human gut ecosystem, and consequently human health, in both beneficial and detrimental ways. In order to understand the role of individual protozoan species in the gut ecosystem, we first need to develop efficient and reliable methods of detection. Here we used metagenomic data to search for ten protozoan genera in eight human populations inhabiting four continents and characterized by contrasted subsistence modes. Our work shows that metagenomics is a promising method for detection and characterization of protozoan assemblages in the gut ecosystem. Comparing different methods of detection (qPCR versus MG for *Blastocystis* sp. and microscopy versus MG for *Entamoeba* spp.), we found no significant difference in sensitivity between qPCR and MG, but a clear improvement in sensitivity between microscopy and MG. However, although qPCR and MG are not significantly different with respect to sensitivity, the biodiversity of *Blastocystis* sp. is better captured by metagenomics. This is quite expected, as qPCR is known to underestimate the prevalence of mixed-subtype colonization because of competitive amplification between differentially abundant STs.

The main advantage of MG is the ability to simultaneously detect a potentially unlimited number of eukaryotic symbionts within a single sampling and analysis protocol. Still, precise and accurate detection of protozoa depends on many factors, among others: sampling and library preparation procedure [40], sequencing depth and coverage [41], community structure [42] as well as the bioinformatic approach [43–45]. Recently, Beghini et al. [24] analyzed the prevalence of *Blastocystis* sp. from published metagenomic data in the same cohort of Tanzanian [26] and Peruvian [25] individuals as we did. Their estimates were considerably lower than ours: 55.6% vs. 81.5% in Tanzania and 16.7% vs. 55.6% in Peru (including all individuals, adults and children). Unlike Beghini et al. [24], we have also found ST8 in USA and ST3 in Italy, as well as a considerable portion of mixed-subtype colonization, with an especially high prevalence in the Tanzanian population (S1, S2 and S4 Figs).

There is a number of differences between our and Beghini et al. [24] analytical procedure that might account for the differences in the prevalence estimates. Firstly, we handled the problem of contamination of publicly available genome assemblies differently. Beghini et al. [24] mapped the available *Blastocystis* genomes to 55 archaeal and bacterial genomes and estimated that 1–30% of nucleotides therein were of bacterial/archaeal origin. They removed these contaminated contigs prior to mapping, whereas we removed the contamination after the alignment step. Secondly, they performed global alignment, whereas we performed local alignment with subsequent filtering of short and low-quality mappings. In addition, Beghini et al. [24] based their prevalence estimates on the breadth of coverage (with a 10% cutoff), while we used a combination of the hit distribution (10% positive contigs) and the breadth of coverage (with a 0.1% cutoff) as our detection criterion. It is noteworthy that applying such a stringent 10% cutoff for breadth of coverage would have resulted in very few positive samples in our case, reflecting our higher stringency in the alignment step. Still, our approach seems to be more sensitive for detection of mixed-subtype colonization, as minor STs are much less likely to be detected with Beghini’s et al. strict cutoff.

Other published MG-based approaches for *Blastocystis* sp. detection include that of Andersen et al. [22], who first constructed co-abundance genes groups (CAGs), and then assigned them to a ST which the majority of the genes in the group aligned to, and that of Forsell et al. [23], who used SSU rRNA gene sequences instead of genomes. In both cases, the power to detect rare STs or mixed colonization of closely related species is considerably reduced. To summarize, intriguingly, none of the previous studies utilizing MG to detect *Blastocystis* sp. found mixed-subtype colonizations [22–24], while these are known to occur [46, 47]. We are thus the first to report mixed *Blastocystis* ST colonization by a metagenomic approach.

DNA-based detection methods, be it PCR or MG, suffer from additional drawbacks in the applied context of detection of enteric pathogens. Namely, a positive result might be due to residual DNA from past infections or to asymptomatic carriage, which commonly occurs in endemic parasite regions [48]. Therefore, careful interpretation is necessary for both diagnostic routine and epidemiological evaluations. Among other challenges for the integration of MG into public health are: high cost, difficulties in comparison of the results from different NGS platforms, rapidly evolving technology and the need for high level of specialized expertise. Although universal bioinformatic approach in the context of public health is yet to be developed [48], the ability of MG to simultaneously address the variation in protozoan and bacterial assemblages will likely enable testing of relevant theoretical and applied questions concerning gut microbiome and human health.

### Prevalence of protoza in the human gut

Out of ten genera we looked for, we detected all of them except *Cryptosporidium* spp., but only three had appreciable (>10%) frequencies: *Blastocystis* sp., *Entamoeba* spp. and *Enteromonas hominis*. As could be expected, we found few pathogenic species (absence of *Cryptosporidium hominis*, *C*. *parvum* and *Entamoeba histolytica*, one individual carrying *Giardia intestinalis* and three individuals carrying *Cystoisospora belli*). Furthermore, we found low prevalence of non-pathogenic species such as *Iodamoeba bütschlii*, *Endolimax nana* and *Chilomastix mesnili*. Similarly, Parfrey et al. 2014 [1], in their SSU rRNA gene amplicon-based study of gut eukaryotic communities, found *Blastocystis* and *Entamoeba*, but no *Chilomastix*, *Cryptosporidium* or *Dientamoeba*.

However, we must consider that the DNA extraction methods used for the generation of the analyzed metagenomic datasets had not been optimized for protozoa, and especially cystic forms of these parasites are known to be often difficult to break (e.g. [49]). Notably, we detected *Cystoisospora belli* and *Dientamoeba fragilis* only in US samples, which were frozen within 24h after collection [25] unlike other samples that were stored in ethanol or ice for a longer time. However, it is known that the storage of stool at 4°C for more than 3 days or at room temperature for one week seriously impedes detection of *D*. *fragilis* [50]. Furthermore, the excretion of vegetative or cystic forms is unpredictable in many protozoan species and intermittent excretion occurs regularly. In addition, SSU rRNA genes can exhibit intraspecific heterogeneity that would result in false negatives given our stringent detection criteria [51]. Nevertheless, despite our stringent cutoffs, we identified more than one protozoan species in 72.5% of infected individuals (S4 Fig). Co-colonization thus seems to be common and often accounts for a large proportion (>50%) of protozoan colonizations detected by traditional methods [18, 52–54]. It is likely that including additional species into the analysis would result in even higher estimates. Similar to other studies [53, 55, 56], we found even the relatively abundant species, such as *Entamoeba coli* or *Enteromonas hominis*, only in mixed colonizations. Therefore, the assembly of the non-pathogenic protozoan community in the gut does not seem to be driven by competition, but rather–as further indicated by the absence of co-exclusions (Fig 4)—by the presence of these protozoa in the environment and the general susceptibility of an individual. The ability of MG to detect mixed infections/colonization in a single analysis could significantly improve worldwide prevalence estimates, but also improve our knowledge about the clinical importance of different protozoa.

### Prevalence of *Blastocystis* sp in worldwide populations

Our results on the prevalence of *Blastocystis* sp. are in line with previous findings, showing that it frequently exceeds 50% in Western, Middle and Eastern African countries [15–19, 57], whereas it ranges from around 35% in urban [58] to over 50% in rural areas of South America [59]. The relatively low prevalence found among the fishers from Cameroon (52.6%) compared with the neighboring hunter-gatherer (81.3%) and farmer populations (95.5%) could reflect the difference in sanitary and environmental conditions. Namely, this group represents a coastal population that lives close to the main road (thus having better access to medical care) and in a less tropical environment than the neighboring hunter-gatherer and farmer populations. A corresponding difference in prevalence has also been observed for *Entamoeba* and intestinal worms [3].

Regarding the individual STs, the absence of ST6, ST7 and ST9, the broad geographic distribution of ST3, ST1 and ST2, as well as the narrow ST4 and ST8 distribution corroborate the results of previous studies [15–19, 24]. Our results provide further evidence for the virtual absence of ST4 from Africa and South America and the sporadic prevalence of ST8 in humans in general [16, 18, 24, 59, 60].

Although we found the prevalence of ST1, ST2 and ST3 to exceed 10% in each of the non-industrialized populations, their distribution varied considerably across countries and lifestyles (Fig 2). Beghini et al. [24] argued that ST2 was strongly affected by industrialization based on its dominance in non-industrialized countries (Peru and Tanzania) and its absence from Europe and USA. Our results corroborate the dominance of ST2 in Tanzanian population, whereas for Peruvian population, we found ST2 to be only the third most prevalent ST. It is noteworthy that other, PCR-based studies of *Blastocystis* sp. STs in South America found a co-dominance of ST1, ST2 and ST3 in rural populations [59], whereas the prevalence of ST2 was considerably lower in urban communities [58]. Similar to Peru, ST2 is only the third most prevalent ST in Cameroon, with considerable (31.3%) prevalence only among hunter-gatherers. The difference in prevalence of ST2 between hunter-gatherers and farmers and fishers from Cameroon (31.3% versus 4.5% and 0%, resp.) is actually quite impressive given that these groups live in the same environment and in very close proximity. Although the hunter-gatherer subsistence mode might seem conducive to colonization by ST2 if we look at Africa only, the absence of ST2 in Peruvian hunter-gatherers does not support this hypothesis. It seems that the factors influencing ST2 distribution are more complex and cannot be reduced to the large-scale effects of either industrialization or subsistence mode.

### Prevalence of *Entamoeba* spp. in worldwide populations

MG revealed a very high prevalence of *Entamoeba* spp. in non-industrialized populations, as well as very frequent co-colonization of the four detected species of *Entamoeba* (Fig 3, S2 Fig). Prevalence of *Entamoeba* is often studied in children (e.g. [52, 54, 61], as the infection with *E*. *histolytica* considerably contributes to diarrheal burden and childhood mortality [62]. Whereas some studies report little variation in prevalence of *Entamoeba* with age [63], others find the contrary [59, 64, 65] and thus the results of these studies may not be directly comparable with ours. However, with few exceptions [52, 54], the reported prevalence is usually much lower (< 25%, [59, 61, 63–66]) than what we have found. Although the discrepancy could be explained by lower sensitivity of microscopy in some cases [63, 65], Ouattara et al. [54] found *E*. *coli* in 62% of examined children in Cote d'Ivoire using this method, possibly reflecting high parasite burden. Regarding the PCR-based studies, the most common species in our study—non-pathogenic *E*. *hartmanni* is less frequently searched for [54, 64] than the *E*. *dispar/histolytica/moshkovskii* complex [52, 54, 59, 64, 66], which could partially explain the observed difference. Still, both Ouattara et al. [54] and Calegar et al. [64] report *E*. *hartmanni* prevalence < 5%, much lower than detected here. The prevalence of another common non-pathogenic *Entamoeba* in our study, *E*. *coli*, seems to vary widely both with geography and subsistence mode, as illustrated by the difference between Tanzania and Cameroon here (Fig 3, S3 Table), a very high prevalence in Cote d'Ivoire [54] and a 5%-25% prevalence in rural South American populations [59, 64, 65]. *E*. *coli* was also a dominant *Entamoeba* (23%) in poor Indian communities [63]. Although our estimate of *E*. *coli* prevalence in Peru (44.4%) is much higher than usually reported from South America [59, 64, 65], a closer look reveals that this is due to a very high prevalence in hunter-gatherers (Fig 3), who were not included in the previous studies. On the other hand, the prevalence in Peruvian farming population corresponds well with the previous reports [59, 64, 65].

Regarding the *E*. *dispar/histolytica/moshkovskii* complex, the absence of pathogenic *E*. *histolytica* is expected given that the individuals were overall healthy. In all populations, we found more *E*. *dispar* than *E*. *moshkovskii*, which we detected only in Africa and never alone (Fig 3). Similarly, Ali et al. [61] reported that the majority (74%) of *E*. *moshkovskii* cases in children in Bangladesh were mixed colonization. Conversely, in rural Columbia, López et al. [52] found app. 25% prevalence of each *E*. *dispar* and *E*. *moshkovskii* and only 5% mixed colonization. HIV-positive Tanzanians actually carried more *E*. *moshkovskii* (13%) than *E*. *coli (*5%, [66]). In the arid areas of Brazil, Calegar et al. [64] found only *E*.*dispar*, but no *E*.*moshkovskii*. These contrasting results highlight the need for better understanding of the ecology of individual *Entamoeba* species and of the complex geographical and cultural factors influencing their distribution.

It is important to note that grouping according to subsistence mode does not reflect fine-scale cultural and environmental differences that may be crucial for understanding of gut protozoa colonization and prevalence in the studied populations. For example, most protozoa are transmitted through fecal-oral route and detailed knowledge about sanitary conditions would likely improve our understanding of the observed prevalence patterns. However, the subsistence mode is likely to influence sanitary conditions to a certain extent, and we therefore think it is a valuable information to account for in the absence of the more detailed data.

## Conclusion and perspectives

Our study is the first to use a metagenomic approach to determine the prevalence of gut protozoa other than *Blastocystis* sp. Specifically, we used MG data to examine the prevalence of ten protozoan genera in eight populations across the globe. The particular approach we applied enabled us to detect high prevalence of mixed colonization in healthy individuals from non-industrialized populations, including both closely and distantly related protozoa. Our results support the hypothesis that protozoa could be common residents of the healthy human gut [2], but also highlight the pitfalls of MG-based diagnostics due to variety of strategies that can be applied to the analysis of MG data. The role of MG in diagnostics will likely become more important with the increasing number of good-quality genomes in the public databases and the development of well-defined diagnostic protocols.

Unveiling the factors that influence the assembly of gut protozoan communities and elucidating their interactions with the human host and its bacterial microbiota are an important step in understanding the gut protozoa role in human health and disease. We first need to better understand drivers of protozoa (co-)colonization by collecting detailed sanitary, medical, anthropological, dietary and other cultural and biological information about the individuals in addition to the sequencing data, as well as describe correlations between these factors. Further, MG-data provide an excellent opportunity to study the effect of gut protozoa on the gut bacterial microbiome diversity as well as its effect on human health. Few such studies exist, but accumulation of metagenomes from non-industrialized populations with higher gut protozoa prevalence should increase their number in the future.

Finally, controlled experiments with vertebrate model organisms will be necessary in order to infer causality and fully elucidate the mechanisms and factors that affect ecological dynamics of individual gut protozoa as well as their communities and interactions with bacterial microbiota and the human host.

## Supporting information

S1 Table Information about the samples used in the study including per-sample results after filtering of the multi-mapped reads. (XLSX)

S2 Table List of protozoan genomes and SSU sequences used for mapping of metagenomic data. (XLSX)

S3 Table Prevalence of detected protozoan species in different populations. (XLSX)

S4 Table Logistic regression models: effect of country and subsistence mode on *Blastocystis* prevalence in non-industrialized countries. (XLSX)

S5 Table Logistic regression models: effect of country and subsistence mode on *Entamoeba* prevalence in non-industrialized countries. (XLSX)

S6 Table Logistic regression models: effect of country and subsistence mode on *Enteromonas* prevalence in non-industrialized countries. (XLSX)

S1 Fig Distribution of *Blastocystis* sp. subtypes within individuals. (PDF)

S2 Fig Distribution of *Entamoeba* species within individuals. (PDF)

S3 Fig Prevalence of *Enteromonas hominis* across populations. (PDF)

S4 Fig Coinfection patterns including all protozoan species. (PDF)

S5 Fig Distribution of mapped reads along genome scaffolds/contigs. (PDF)
