# Supplementary material for: Use of shotgun metagenomics for the identification of protozoa in the gut microbiota of healthy individuals from worldwide populations with various industrialization levels
Source: PLoS One. 2019 Feb 6;14(2):e0211139. doi: 10.1371/journal.pone.0211139 (PMC6364966; doi:10.1371/journal.pone.0211139)
Supplement: S1 Fig — (PDF) [file pone.0211139.s007.pdf]

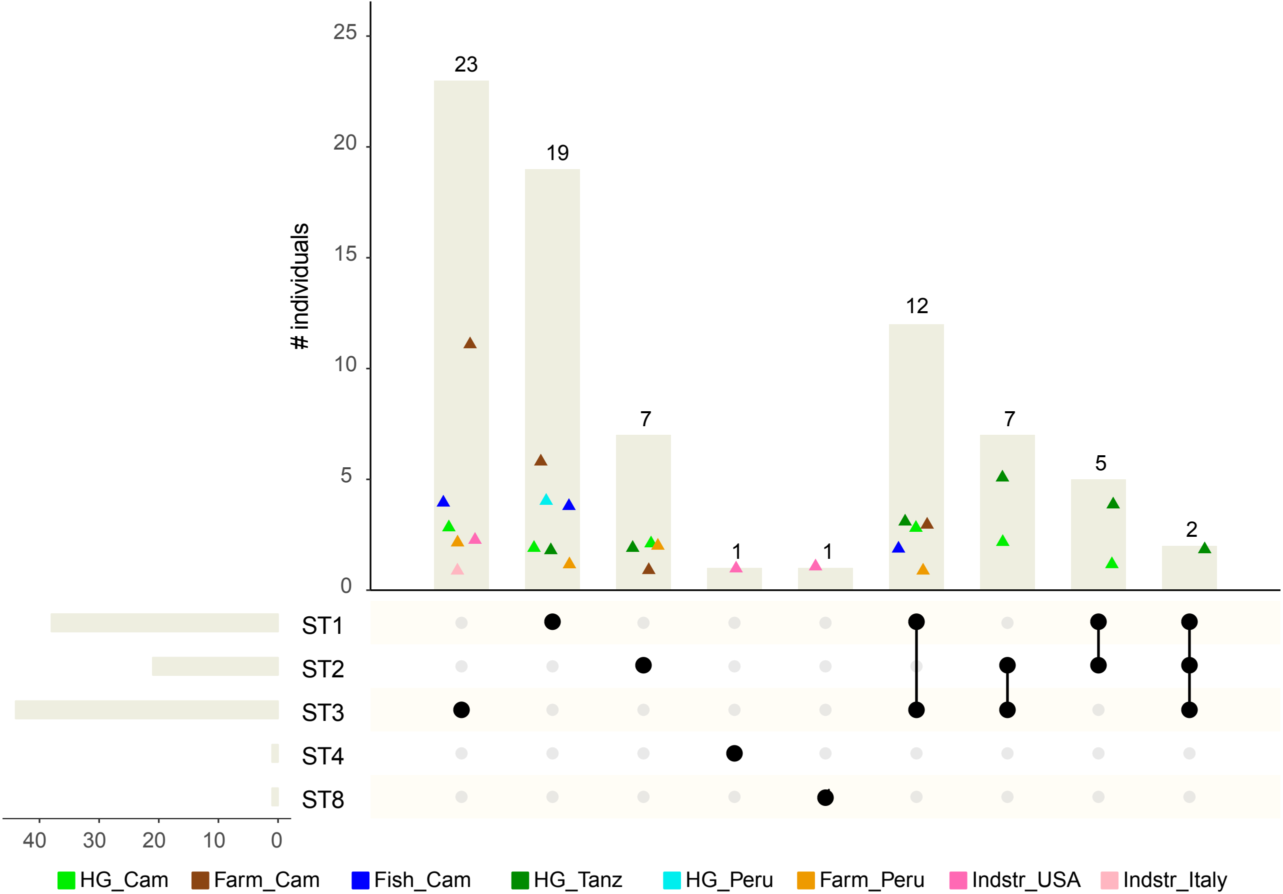

S1 Fig. Distribution of *Blastocystis* sp. subtypes within individuals. Triangles denote number of subjects in a population with a given coinfection pattern. Only subtypes with non-zero prevalence are shown.
