# Supplementary material for: Use of shotgun metagenomics for the identification of protozoa in the gut microbiota of healthy individuals from worldwide populations with various industrialization levels
Source: PLoS One. 2019 Feb 6;14(2):e0211139. doi: 10.1371/journal.pone.0211139 (PMC6364966; doi:10.1371/journal.pone.0211139)
Supplement: S2 Fig — (PDF) [file pone.0211139.s008.pdf]

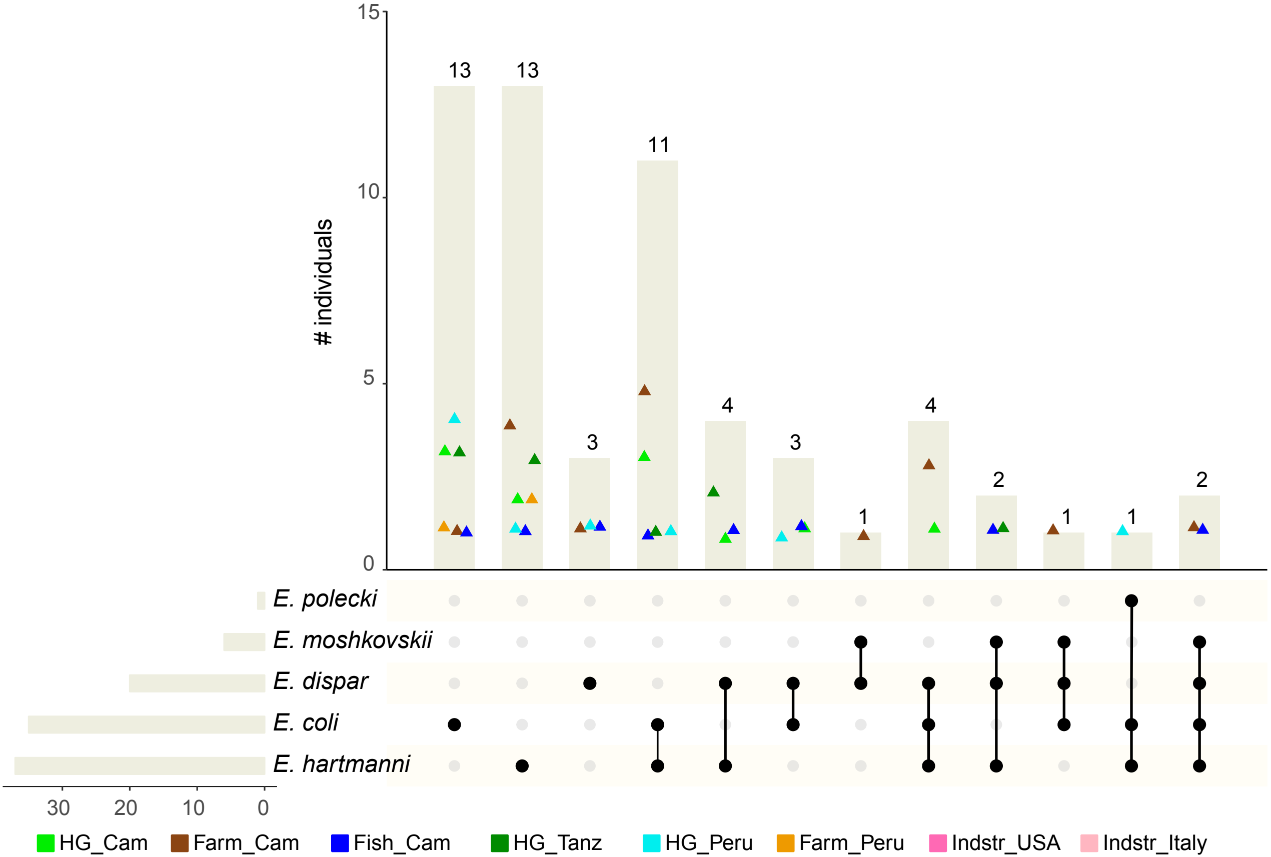

S2 Fig. Distribution of *Entamoeba* species within individuals. Triangles denote number of subjects in a population with a given coinfection pattern. Only species with non-zero prevalence are shown.
