# Supplementary material for: Use of shotgun metagenomics for the identification of protozoa in the gut microbiota of healthy individuals from worldwide populations with various industrialization levels
Source: PLoS One. 2019 Feb 6;14(2):e0211139. doi: 10.1371/journal.pone.0211139 (PMC6364966; doi:10.1371/journal.pone.0211139)
Supplement: S3 Fig — (PDF) [file pone.0211139.s009.pdf]

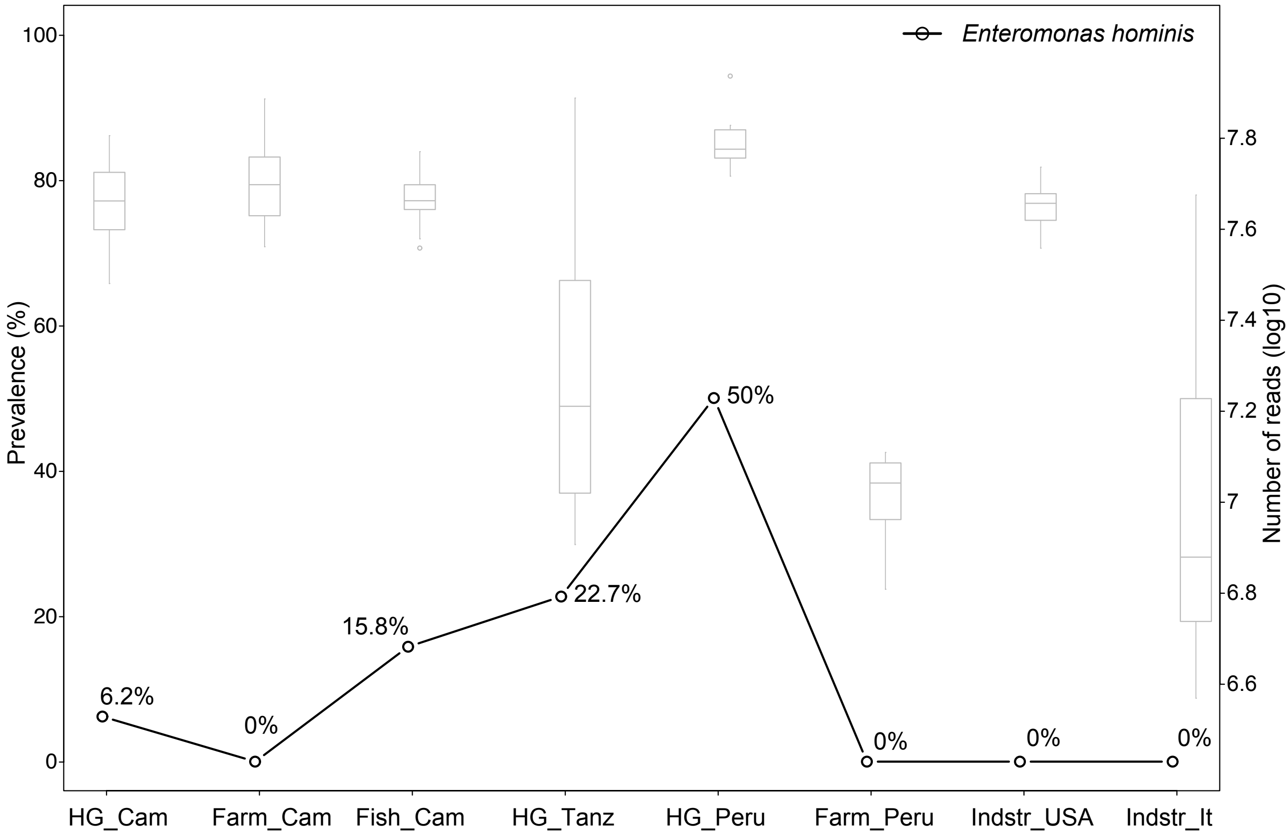


**S3 Fig. Prevalence of *Enteromonas hominis* across populations.** The population abbreviations are the same as in Fig 2. Grey boxplots show the average number of filtered reads per sample (right Y-axis).
