# Supplementary material for: Use of shotgun metagenomics for the identification of protozoa in the gut microbiota of healthy individuals from worldwide populations with various industrialization levels
Source: PLoS One. 2019 Feb 6;14(2):e0211139. doi: 10.1371/journal.pone.0211139 (PMC6364966; doi:10.1371/journal.pone.0211139)
Supplement: S5 Fig — (PDF) [file pone.0211139.s011.pdf]

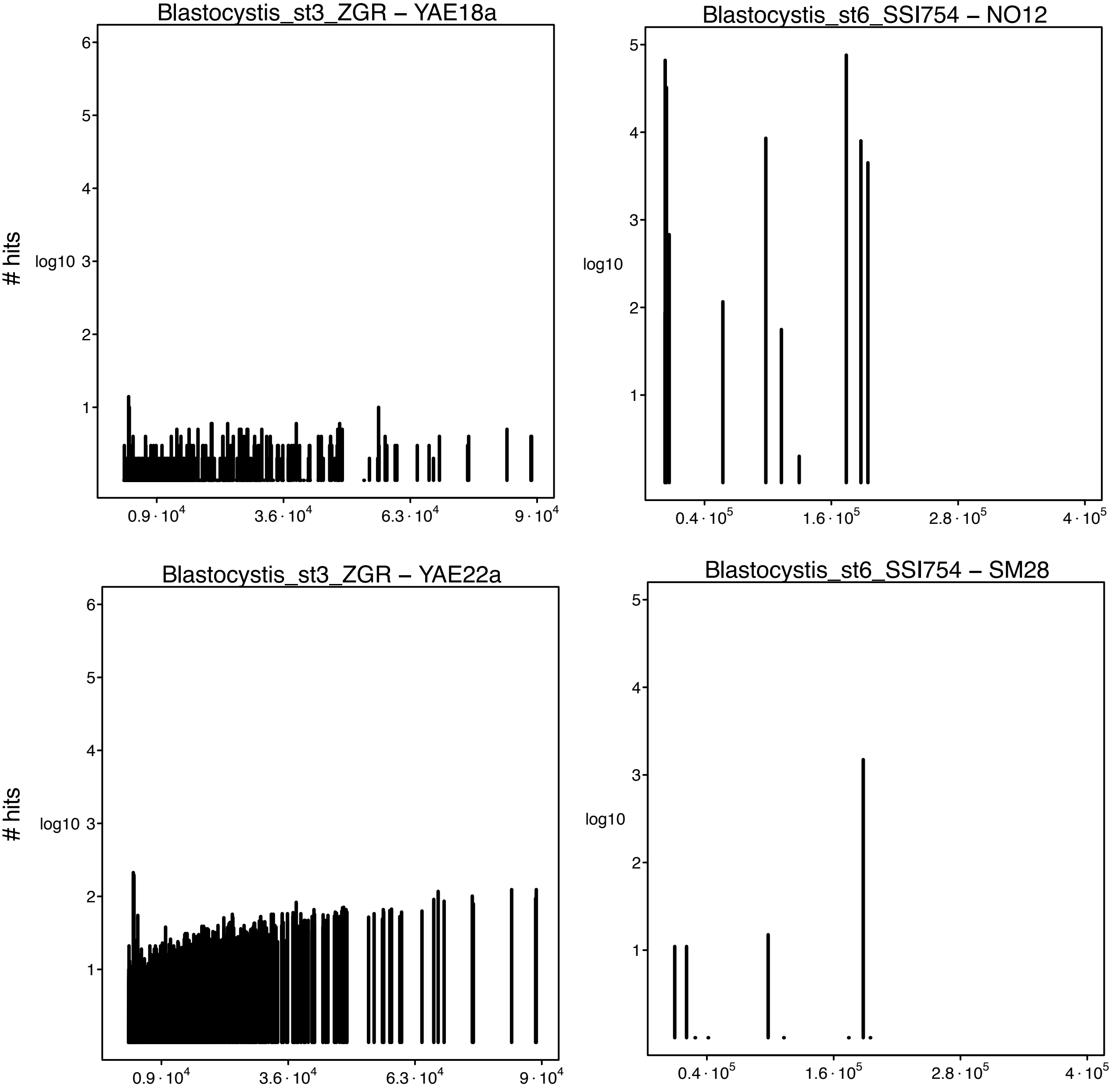

S5 Fig. Distribution of mapped reads along genome scaffolds/contigs. Left column shows examples of "good" distribution with the hits more or less uniformly distributed across scaffolds. Right column shows "bad" distribution, with the hits concentrated in few regions.
